# Sepsis and neutropenia in hematological malignancies

**DOI:** 10.1186/cc14050

**Published:** 2014-12-03

**Authors:** Y Labiad, L Farnault, B Loriod, R Costello, C Nguyen

**Affiliations:** 1 Technological Advances for Genomics and Clinics U1090, INSERM, Marseille, France; 2 Service d'hématologie de La Conception, Assistance publique - Hôpitaux de Marseille, France

## Introduction

The pre-existing inflammatory state influences the behavior of infection, a first peak of inflammation may be the risk factor during infection and predispose to a severe form of the disease [1,2]. This hypothesis is frequently accepted but has never been tested. Patients with hematological malignancies treated by chemotherapy in case of autologous stem cell transplantation (ASCT) are exposed to a neutropenic state: an abnormal low number of neutrophils. In the neutropenic patient, sepsis is more frequent than in the non-neutropenic patient. The severity of infection depends on many factors, particularly the pre-existing inflammatory state, the genetic factors, and the duration and the depth of neutropenia. In the literature, the factors involved in inflammation and infection are often confused. On the other hand, no predictive transcriptomic signature of sepsis was found. Our objective is to identify predictive genes that influence the outcomes of the infection in neutropenic patients.

## Methods

To test our hypothesis, high-throughput transcriptomic analysis and bioinformatics will be combined, laying on the modulation of gene expression of the peripheral blood mononuclear cells of patients with hematological malignancies treated by chemotherapy in case of ASCT.

## Results

A preliminary study on a reduced number of patients has allowed us to assess the feasibility of the project. After a statistical analysis, 615 genes are differently expressed between patients who developed sepsis and those who have not developed with a false discovery rate of 5%. These 615 genes are potentially predictive of sepsis 2 days before the development of the infection. Furthermore, 28 genes are differentially expressed between patients who developed sepsis and those who have not developed with a false discovery rate of 5%, and these 28 genes are potentially predictive of sepsis 7 days before the development of the infection.

## Conclusion

The ongoing transcriptomic study with a larger effect will allow us to test our hypothesis and confirm the results of our preliminary study. The microarray results should be strengthened with quantitative PCR assays, than validated with protein assays. Finally, we will transfer the experimental data to clinical practices.

